# Environmental Factors Associated with African Swine Fever Persistence in Wild Boar Populations in Lithuania

**DOI:** 10.3390/ani16213439

**Published:** 2026-10-30

**Authors:** Gabija Džiaugienė, Katja Schulz, Marius Masiulis, Carola Sauter-Louis, Alvydas Malakauskas

**Affiliations:** 1 Department of Veterinary Pathobiology, Veterinary Academy, Lithuanian University of Health Sciences, 47181 Kaunas, Lithuania; alvydas.malakauskas@lsmu.lt; 2 Institute of Epidemiology, Friedrich-Loeffler-Institut, 17493 Greifswald-Insel Riems, Germany; katja.schulz@fli.de (K.S.);; 3 Dr. L. Kriaučeliūnas Small Animal Clinic, Faculty of Veterinary Medicine, Veterinary Academy, Lithuanian University of Health Sciences, 47181 Kaunas, Lithuania

**Keywords:** African swine fever, wild boar, endemicity, environmental factors, forest cover, wetlands, carcass detection, spatial epidemiology, Lithuania

## Abstract

Since 2014, Lithuania has been affected by African swine fever (ASF), with varying levels of disease occurrence. This study investigated factors associated with ASF persistence in wild boar populations across Lithuanian municipalities. Based on ASF occurrence patterns, municipalities were classified into three groups: endemic, non-endemic, and ‘no ASF detected’. Endemic municipalities had ASF cases reported for at least three consecutive years. Data from 2016 to 2022 were analysed, including numbers of wild boar hunted or found dead, environmental variables, and spatial characteristics. Analyses included non-parametric tests, correlation analyses, negative binomial regression, and longitudinal logistic regression using generalised estimating equations to assess associations with ASF occurrence. Differences were observed between municipality groups, with endemic areas showing higher numbers of ASF cases and found dead wild boar. The number of hunted wild boar was positively correlated with the number of ASF-positive hunted wild boar, likely reflecting hunting-related sampling effort. Forest cover was negatively associated with ASF case counts, while wetland coverage was positively associated with endemic status, case counts and ASF presence over time. The findings highlight the importance of surveillance strategies, especially those based on carcass detection and testing. Environmental factors, including wetland-associated conditions, may influence ASF occurrence. Results support the need for targeted, landscape-informed approaches to ASF surveillance and control in endemic regions.

## Simple Summary

Since 2014, Lithuania has been affected by African swine fever (ASF), with varying levels of disease occurrence across regions. This study aimed to identify factors associated with the persistence of ASF in wild boar populations at the municipality level. Municipalities were classified as endemic, non-endemic, or with no ASF detected, based on whether the disease was detected for at least three consecutive years. The analysis covered the period from 2016 to 2022 and identified differences in ASF occurrence between municipality groups. The number of hunted wild boar did not differ significantly between groups, suggesting that hunting numbers alone were not sufficient to explain differences in disease occurrence. The high proportion of ASF-positive animals among found dead wild boar supported the importance of carcass detections and passive surveillance for identifying areas with ongoing virus circulation. Environmental factors were also associated with ASF detection patterns. Higher forest cover was linked to fewer detected ASF cases, whereas wetland areas were associated with a higher probability of ASF occurrence. These findings suggest that environmental conditions may contribute to virus persistence. Overall, this study supports targeted surveillance, including carcass search, and indicates that environmental conditions should be considered when managing ASF in wild boar populations.

## 1. Introduction

Since its introduction in Georgia in 2007, African swine fever (ASF) has spread rapidly among wild boar and domestic pig populations across Europe and remains a significant challenge for animal health authorities in the European Union [1]. The ASF virus (ASFV), which infects only suids, often causes case fatality rates exceeding 95% in both domestic pigs and wild boar [2]. Consequently, in affected regions, the disease has a severe impact on pork production and international trade, and causes substantial economic losses for the agricultural and hunting sectors. Over the past decade, ASF has affected numerous European countries and has become one of the most important transboundary animal diseases threatening pig production systems in Europe. Wild boar populations are considered the main reservoir and driver of ASFV circulation in many affected regions, significantly complicating disease control and elimination efforts [1].

While ASF outbreaks in domestic pigs can often be prevented through strict biosecurity measures, the situation in Eurasian wild boar populations is more complex. Field observations from several affected European countries have shown that, once introduced into wild boar populations, ASFV may persist for extended periods and become endemic [3,4]. Despite the high lethality caused by ASF and its potential to substantially reduce wild boar populations, virus circulation is not necessarily interrupted [5]. As a result, infected wild boar populations remain an important source of infection and continue to challenge long-term disease control.

ASFV transmission in wild boar occurs through both direct contact between animals and indirect contact with contaminated carcass material or the surrounding environment. The virus can remain infectious in carcasses and contaminated habitats for prolonged periods, thereby contributing to continuous circulation. Wild boar have been observed coming into contact with carcasses of conspecifics, creating opportunities for indirect transmission [6,7], and ASFV stability in carcass material may further support carcass-mediated transmission [8]. ASFV DNA has also been detected in contaminated soil after carcass decomposition or removal, suggesting that environmental contamination may contribute to virus persistence [9]. Together, these findings support the concept of the wild boar-habitat cycle, in which infected carcasses and contaminated habitats contribute to long-term virus persistence and complicate eradication efforts [10].

Although considerable progress has been made in understanding ASFV characteristics and transmission pathways [11], detailed knowledge regarding the factors that support the long-term persistence and endemicity of the disease in wild boar populations remains limited. Most studies have focused on transmission dynamics and short-term spread, while factors influencing long-term persistence at the regional level remain less clearly understood. Observations from different regions indicate that ASF dynamics may vary considerably: in some areas the virus persists within local wild boar populations for prolonged periods, whereas in others it seems to disappear after a relatively short time [12]. These differences suggest that a combination of ecological, epidemiological, environmental, and control-related factors may influence the ability of the virus to persist locally for extended periods of time. Several studies have investigated potential drivers of ASF persistence and spread in wild boar populations, including host density, movement ecology, landscape structure and environmental conditions influencing virus circulation [11,13]. However, the relative importance of these factors may vary between regions, and the mechanisms supporting long-term virus persistence remain insufficiently understood. A better understanding of these factors can support improvements in ASF surveillance strategies and the development of more effective disease control measures in endemic areas to achieve elimination of ASF.

The first cases of ASF in wild boar in Lithuania were reported in the south-eastern part of the country in January 2014, marking the beginning of a persistent threat to the national pig sector [14,15]. Since then, the disease has spread westwards across the country, with considerable variation in ASF prevalence among hunted and found-dead wild boar [12,16]. In response, extensive surveillance and control measures were implemented, including intensified passive surveillance, changes in hunting strategies, and strengthened biosecurity requirements for pig holdings.

Despite these efforts, ASFV continues to circulate in wild boar populations across the entire territory of Lithuania. Over more than a decade, heterogeneous epidemiological patterns have been observed, including areas with recurring outbreaks and regions where the virus persists for extended periods.

These regional differences provide an opportunity to investigate factors influencing ASF persistence. Therefore, this study aims to identify environmental factors associated with the endemic persistence of ASF in wild boar populations across Lithuanian municipalities.

## 2. Materials and Methods

### 2.1. Data

The study period covered January 2016 to December 2022. Wild boar data originated from the CSF/ASF wild boar surveillance database of the European Union (https://surv-wildboar.eu, accessed on 1 October 2023). The database contains national surveillance data submitted by the State Food and Veterinary Service of Lithuania, the competent veterinary authority. The dataset included data on wild boar that were hunted, found dead, shot due to illness, or involved in road traffic accidents. Wild boar shot due to illness were included in the hunted category, whereas animals involved in road traffic accidents were excluded from the analysis. For each animal, the date of detection (or sample registration) and geographic coordinates were recorded when available.

During 2016–2022, all wild boar carcasses in Lithuania were tested for ASFV using polymerase chain reaction (PCR). Data from this period were used because the testing strategy remained unchanged throughout these years. The total number of hunted wild boar also represented the number tested and was used as a proxy for hunting-related sampling effort. Detailed information on the samples tested can be found in Pautienius et al. [4].

Additional data on ASF outbreaks in domestic pigs at the municipality level were obtained from the State Food and Veterinary Service of Lithuania. Human population data at the municipality level were retrieved from the Official Statistics Portal of Lithuania (osp.stat.gov.lt, accessed on 25 January 2024).

Lithuania is administratively divided into 60 municipalities, of which 50 were included in the analysis. Three of these municipalities were city or resort-type municipalities, with substantial wild boar populations. All other city municipalities (*n* = 7) were excluded due to their limited wild boar presence. In addition, three municipalities (Kretinga, Pagegiai, and Silale) were excluded because ASF cases were reported only in the final two years of the study period. These municipalities could not yet meet the three-consecutive-year criterion for endemic status, but classifying them as non-endemic would have been premature, as detections might have continued beyond the study period.

### 2.2. Spatial Variables

Spatial data processing and analysis were performed using QGIS software (version 3.32.2). All spatial data were projected to the Lithuanian national coordinate system (EPSG:3346—LKS94/Lithuania TM).

Municipality boundaries were obtained from EuroGeographics (EuroGlobalMap). Minor topological adjustments were made where necessary to ensure consistency of administrative units for spatial analysis.

Forest cover data were derived from the Global Forest Change dataset (version 1.12; [17]). Pixels with ≥30% canopy cover in the year 2000 were classified as forest. Annual forest cover for the period 2016–2022 was reconstructed by accounting for cumulative forest loss. Zonal statistics were used to calculate forest area within each municipality, which was then expressed as a percentage of total municipality area.

Wetland and river data were obtained from the Statistical Department of Lithuania. Wetland coverage was calculated by intersecting spatial datasets of wetlands with municipality boundaries. For each municipality, total wetland area was calculated and expressed as a percentage of the total municipality area. River density was calculated as the total river length (km) per unit area (km^2^) within each municipality.

### 2.3. Grouping and Outcome

Municipalities were classified into three groups based on ASF occurrence patterns: endemic, non-endemic, and with no ASF detected. A municipality was defined as endemic if ASF cases were reported for at least three consecutive years during the study period. The three-year threshold was selected as a pragmatic operational criterion, informed by epidemiological experience, to distinguish prolonged ASF occurrence from shorter periods of detection. Municipalities with no reported ASF cases throughout the entire study period were classified in the ‘no ASF detected’ group, while the remaining municipalities were categorised as non-endemic.

The primary outcome variable was the number of ASF-positive wild boar cases per municipality per year. For specific analyses, a binary outcome variable indicating the presence (1) or absence (0) of ASF cases in a municipality per year was also used.

### 2.4. Statistical Analysis

Statistical analysis was performed using IBM SPSS Statistics (version 29.0.0.0). Normality of variables used in group comparisons was assessed using the Shapiro–Wilk test. As most variables were not normally distributed, non-parametric methods were used for group comparisons. The variables included in the statistical analyses, their definitions, and their use in the analyses are summarised in Table 1.

Differences between municipality groups (endemic, non-endemic, and ‘no ASF detected’) were assessed using the Kruskal–Wallis H test. Where appropriate, pairwise comparisons were performed using the Mann–Whitney U test with Bonferroni adjustment.

Associations between municipality ASF status and border-related categorical variables were evaluated using the Chi-square test of independence. Two border-related variables were assessed: country border presence, defined as whether a municipality had an international border (yes/no), and border country, defined as the name of the neighbouring country or no border.

Spearman’s rank correlation coefficient was used to assess relationships between continuous variables, including the number of ASF cases and the number of hunted wild boar.

Univariable binary logistic regression was used to compare endemic and non-endemic municipalities, with endemic status as the dependent variable and forest cover and wetland coverage tested as independent variables in separate models.

To model ASF case counts, a generalised linear model (GLM) with a negative binomial distribution and a log link function was applied due to overdispersion in the data. The dependent variable was the annual number of ASF-positive cases per municipality. Independent variables included annual forest cover (%), surveillance year (2016–2022), and the log-transformed number of hunted wild boar. Study year was treated as a categorical factor, and the natural logarithm of municipality area was included as an offset variable. In addition, a multivariable negative binomial model including both forest cover (%) and wetland coverage (%) was fitted to assess whether both environmental variables remained associated with ASF-positive case counts when included in the same model. The negative binomial models included annual observations for each municipality and adjusted for study year as a categorical factor. Within-municipality correlation across years was not explicitly modelled in these analyses.

To evaluate the association between environmental variables and ASF occurrence over time, a longitudinal logistic regression model using generalised estimating equations (GEE) was applied. The binary outcome variable represented ASF presence (1) or absence (0) per municipality per year. Forest cover and wetland coverage were tested as environmental predictors in separate models. Municipality name was used as the subject variable and year as the within-subject variable. An independent working correlation structure was used, and models were adjusted for surveillance year and the log-transformed number of hunted wild boar as a proxy for hunting effort.

The models addressed distinct outcomes: endemic status represented municipality-level classification over the study period; annual case counts measured the number of detected ASF-positive wild boar per municipality-year; and annual presence/absence indicated whether at least one positive animal was detected in a municipality in a given year. Associations with these outcomes may therefore differ, as they describe different aspects of ASF detection.

For each surveillance category, annual PCR positivity percentages were calculated using the number tested as the denominator, with 95% confidence intervals calculated using the Wilson score method.

### 2.5. Ethical Approval

Ethical approval for this study was granted by the Bioethics Centre of the Lithuanian University of Health Sciences (LUHS) (No. 2026-BEC3-T-082). The study was based on epidemiological data and did not involve direct animal experimentation.

## 3. Results

### 3.1. Descriptive Overview

During the study period (2016–2022), a total of 143,808 wild boars were tested for ASFV in the 50 municipalities included in the analysis. Of these, 137,480 (95.6%) were hunted wild boar and 6328 (4.4%) were found dead wild boar. Overall, 5365 samples tested positive by PCR, corresponding to a detection rate of 3.7%. Annual numbers of hunted and found dead wild boar tested for ASFV, together with ASF-positive counts and percentages with 95% confidence intervals by surveillance category, are provided in Appendix A, Table A1.

**Figure 1:**
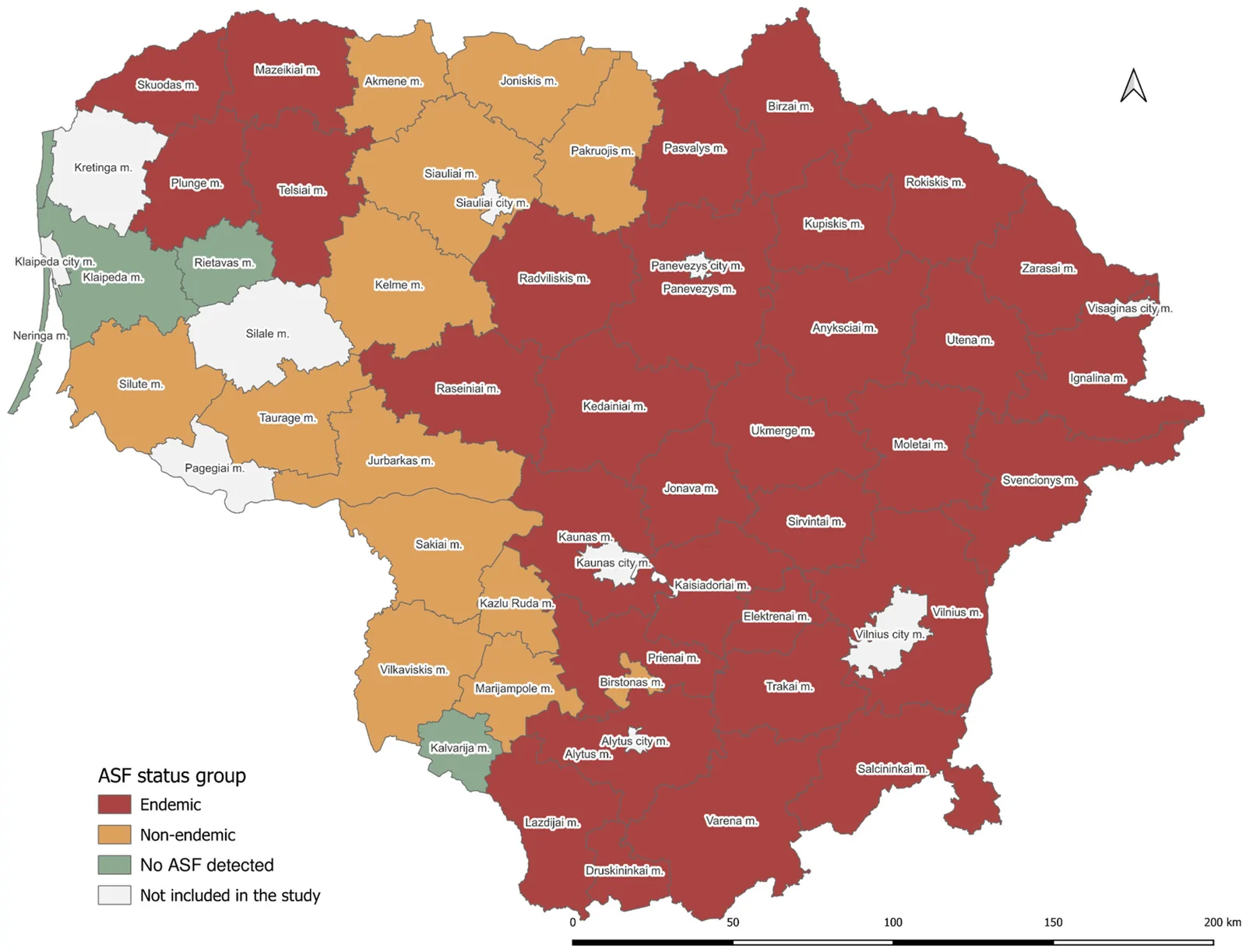
Distribution of Lithuanian municipalities by ASF status group during 2016–2022.

Municipalities were classified into three groups: endemic (*n* = 32), non-endemic (*n* = 13), and ‘no ASF detected’ (*n* = 5). ASF-positive cases peaked in 2017–2018 and declined thereafter, with lower and more sporadic detection patterns observed in non-endemic municipalities. The spatial distribution of municipalities in the endemic, non-endemic, and ‘no ASF detected’ groups is shown in Figure 1. Municipality area, ASF status group, total number of ASF-positive wild boar cases, and ASF-positive case density are provided in Appendix A, Table A2.

### 3.2. Group Differences

**Figure 2:**
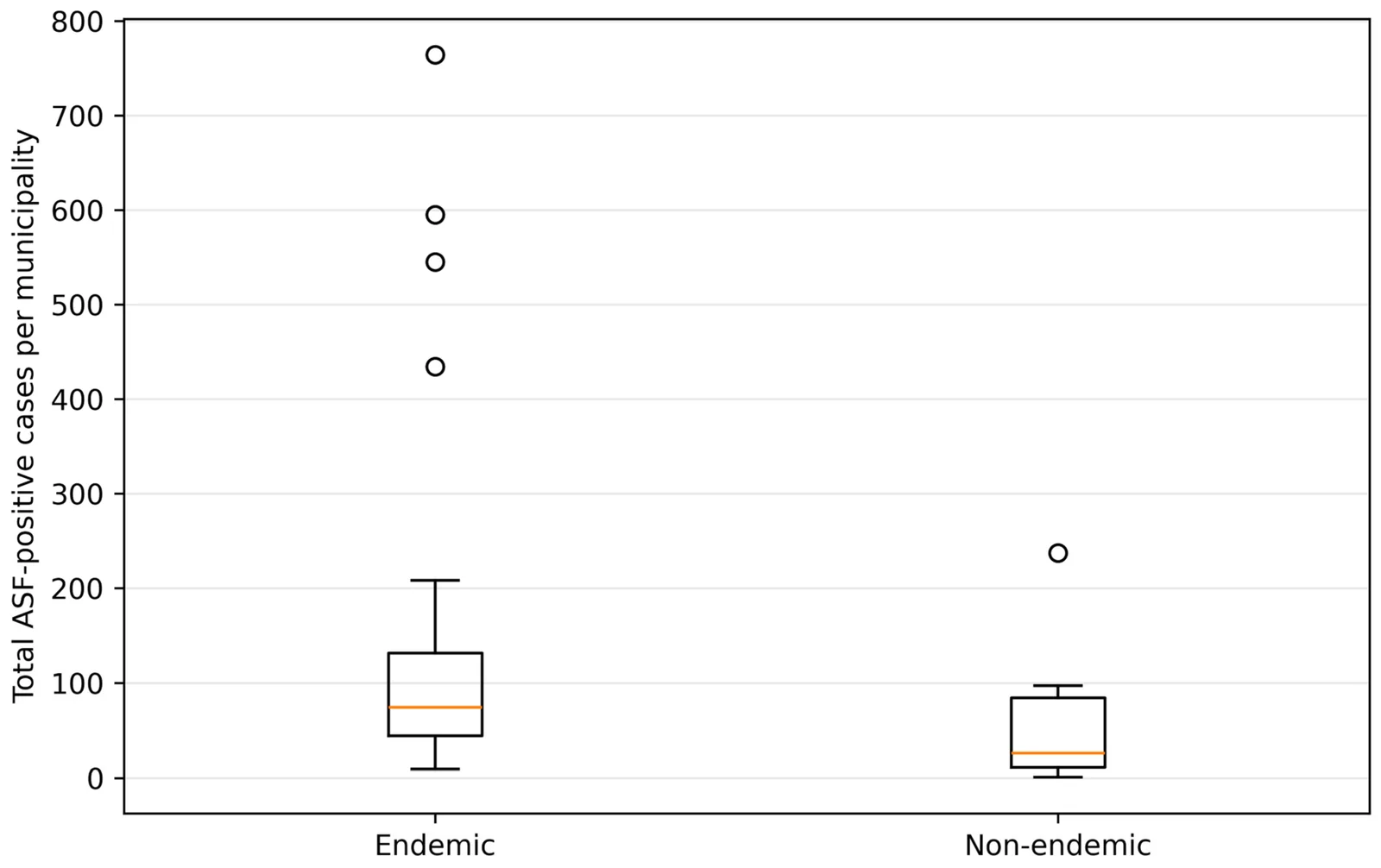
Distribution of total ASF-positive wild boar cases in endemic (*n* = 32) and non-endemic (*n* = 13) municipalities in Lithuania during 2016–2022. Boxes indicate the interquartile range, orange lines indicate medians, whiskers show the range excluding outliers, and circles indicate outliers.

A statistically significant difference in the total number of ASF-positive cases per municipality during 2016–2022 was observed between municipality groups (Kruskal–Wallis H test, χ^2^ (df 2) = 18.15, *p* < 0.001). Pairwise comparisons showed that this difference was significant only between endemic municipalities and municipalities with no ASF detected (adjusted *p* < 0.001), whereas endemic and non-endemic municipalities did not differ significantly. The distribution of total ASF-positive cases in endemic and non-endemic municipalities is shown in Figure 2.

A similar pattern was observed for found dead wild boar. Significant differences between municipality groups were found both in the total number of found dead wild boar submitted for ASFV testing (χ^2^ (df 2) = 13.29, *p* < 0.001) and in the number of ASF-positive found dead wild boar (χ^2^ (df 2) = 17.86, *p* < 0.001). In both analyses, pairwise comparisons indicated significant differences only between endemic and municipalities with no ASF detected.

In addition, the proportion of ASF-positive animals among wild boar found dead and tested for ASFV was calculated. Overall, 4493 of 6328 found dead wild boar were ASF-positive (71.0%). This proportion was higher in endemic municipalities (3952/5414; 73.0%) than in non-endemic municipalities (541/873; 62.0%). These percentages are presented descriptively; the difference between groups was not formally tested for statistical significance.

No statistically significant differences were found in the number of hunted wild boar between municipality groups (χ^2^ (df 2) = 1.39, *p* = 0.498).

### 3.3. Correlations

Spearman correlation analysis showed significant positive associations between the annual number of hunted wild boar and ASF-positive hunted wild boar in 2018–2022, with correlation coefficients ranging from 0.29 to 0.56 (*p* < 0.05; Table 2).

Additional exploratory analyses of selected municipality-level variables are provided in Appendix A, Table A3. Human population indicators were not statistically significantly correlated with wild boar ASF case numbers, and river density did not differ statistically significantly between ASF status groups. Same-year domestic pig ASF cases showed positive correlations with wild boar ASF case numbers in several years.

### 3.4. Environmental Factors

Forest cover ranged from 7.5% to 68.9%, with mean values around 30–33% in all years. In the univariable logistic regression comparing endemic and non-endemic municipalities, wetland coverage was statistically significantly associated with endemic municipality status (B = 0.452, OR = 1.57; 95% CI: 1.274–1.939; *p* < 0.001). In contrast, forest cover was not statistically significantly associated with endemic status (B = 0.014, OR = 1.01; 95% CI: 0.996–1.032; *p* = 0.127).

**Figure 3:**
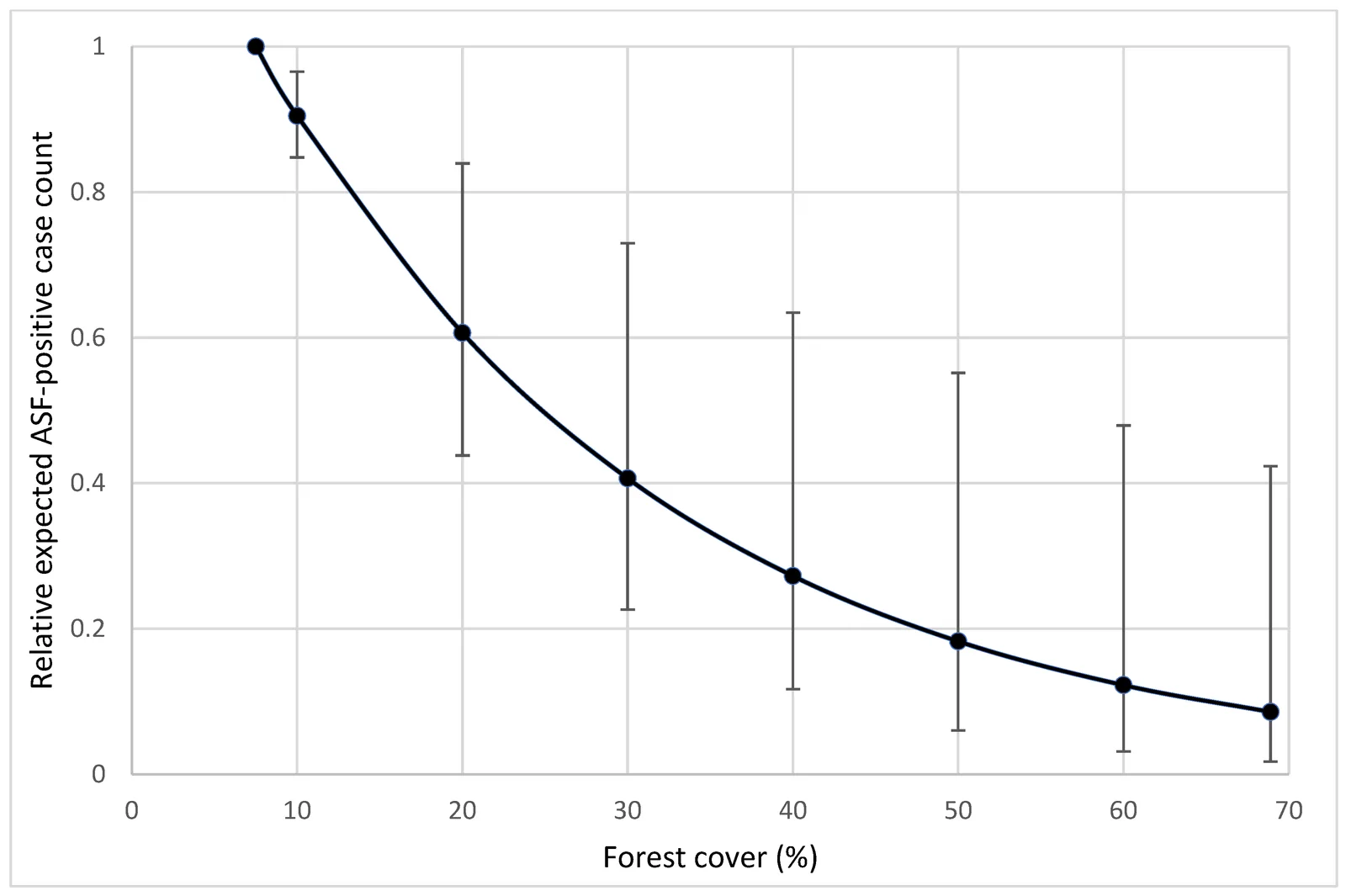
Model-based effect plot showing the association between forest cover (%) and relative expected ASF-positive wild boar case counts. The curve is based on the negative binomial regression coefficient for forest cover (B = –0.040), adjusted for surveillance year and ln(hunted wild boar), with ln(municipality area) included as an offset and the lowest observed forest cover value set as the reference. Vertical error bars indicate approximate 95% confidence intervals. Dots indicate model-based estimates at selected forest cover values.

In the negative binomial regression model, forest cover (%) was statistically significantly negatively associated with ASF case counts (B = −0.040, SE = 0.013, *p* = 0.002), indicating fewer ASF cases in municipalities with higher forest coverage. This model-based association is illustrated in Figure 3. However, in the longitudinal presence/absence analysis, forest cover was not statistically significantly associated with ASF absence (B = −0.025, OR = 0.98; 95% CI: 0.94–1.01; *p* = 0.155). Temporal effects were also statistically significant, with higher case numbers observed in earlier years compared to 2022.

Wetland coverage ranged from 0.04% to 4.13% across municipalities (mean 1.49%, SD = 1.19). Significant differences were observed between municipality groups (Kruskal–Wallis H = 14.40, *p* < 0.001), with higher wetland proportions in endemic areas. In the negative binomial regression model, wetland coverage was significantly positively associated with ASF case counts (B = 0.251, SE = 0.101, *p* = 0.013). The corresponding incidence rate ratio was 1.29 (95% CI: 1.06–1.57), indicating that each one-percentage-point increase in wetland coverage was associated with approximately 29% higher ASF case counts.

When forest cover and wetland coverage were included in the same negative binomial model, both variables remained statistically significant. Forest cover was negatively associated with ASF-positive wild boar case counts (B = −0.043, SE = 0.006, *p* < 0.001), whereas wetland coverage was positively associated with case counts (B = 0.128, SE = 0.053, *p* = 0.017), after adjustment for surveillance year and hunting-related sampling effort, with municipality area included as an offset.

In the longitudinal logistic regression model, wetland coverage was also significantly associated with ASF presence (B = 0.278, OR = 1.32; 95% CI: 1.002–1.739, *p* = 0.048), indicating that each one-percentage-point increase in wetland coverage was associated with approximately 32% higher odds of ASF occurrence. Overall, wetland coverage was consistently associated with ASF occurrence across the count and presence/absence models. A summary of the regression model results is presented in Table 3.

## 4. Discussion

Lithuania has been affected by ASF since 2014. ASF-positive wild boar were repeatedly detected in some areas, whereas detections were sporadic in others.

### 4.1. Key Findings

This study assessed factors associated with ASF persistence in wild boar populations across Lithuanian municipalities. Among the variables analysed, environmental factors showed the strongest associations with ASF detection patterns. Forest cover was negatively associated with detected ASF case counts, whereas wetland coverage was associated with ASF presence. The additional multivariable count model supported these findings, as forest cover and wetland coverage remained associated with ASF-positive case counts when included in the same model. These findings suggest that landscape-related conditions may be relevant for understanding differences in ASF detection and persistence at the municipality level.

Endemic and non-endemic municipalities did not differ statistically significantly in the total number of detected ASF-positive cases, although endemic municipalities were characterised by repeated virus detection over several consecutive years. This suggests that endemic classification in this study did not simply reflect higher cumulative case numbers. Non-endemic municipalities may have experienced shorter periods of more intense virus detection, followed by no further detections in three consecutive years, whereas endemic municipalities were characterised by longer-term continuity of virus detection. This distinction highlights the importance of considering temporal patterns of ASF detection, rather than only the total number of detected cases, when interpreting ASF persistence at the municipality level.

The univariable logistic regression comparing endemic and non-endemic municipalities further supported the relevance of wetland coverage, which was significantly associated with endemic municipality status. In contrast, forest cover was not significantly associated with endemic status in this comparison. However, this finding should be interpreted cautiously because the comparison was based on unequal group sizes and univariable models.

No significant differences in the number of hunted wild boar were observed between municipality groups. This suggests that ASF status was not reflected in a measurable difference in hunting output at the municipality level. The observed patterns might be influenced by a combination of surveillance intensity and environmental factors.

### 4.2. Environmental Factors

The observed associations with forest cover and wetland coverage should be interpreted in the context of previous ASF studies and the municipality-level scale of the analysis. Forested habitats have frequently been identified as high-risk environments for ASF occurrence in wild boar across Europe, mainly because they provide suitable habitat, support wild boar movement, and may facilitate contact with infectious carcasses or contaminated environments [11,18]. Landscape structure and connectivity may further influence ASF spread, as continuous suitable habitats can facilitate dispersal, whereas fragmented landscapes may limit movement and reduce the probability of transmission between local wild boar groups [19,20].

In contrast to previous studies, forest cover was negatively associated with detected ASF case counts in the present analysis. Similar discrepancies have been discussed in previous spatial studies, where associations between forest cover and ASF occurrence were influenced by surveillance intensity, accessibility, and the spatial scale of analysis [18]. However, this association should be interpreted cautiously, as the analysis was conducted at the municipality level and was based on detected cases rather than true infection prevalence. In highly forested municipalities, carcasses may be more difficult to detect, hunting and reporting intensity may differ from that in more accessible areas, and detected case counts may therefore underestimate ongoing virus circulation. In addition, long-term ASF presence and control measures may have reduced local wild boar abundance in some affected forested areas, which could also contribute to lower detected case numbers. Although forest cover was associated with detected case counts, no statistically significant association with annual ASF presence was identified in the longitudinal presence/absence analysis.

Compared with the forest-related results, wetland coverage showed a more consistent association with ASF occurrence and persistence. It was associated with endemic municipality status, higher ASF case counts, and ASF presence over time. In the additional multivariable count model, wetland coverage also remained positively associated with ASF-positive case counts when analysed together with forest cover. Although wetlands represented only a small proportion of municipality area, their association with ASF occurrence is consistent with the hypothesis that moist and water-associated habitats may be relevant for local virus maintenance. Similar patterns have been reported in recent studies assessing environmental risk factors for ASF [21]. Wetland environments may influence wild boar behaviour, carcass persistence, and virus persistence. ASF-infected wild boar have been shown to select cool and moist habitats, often close to water sources, during the final stages of disease [7,21,22]. Such behaviour may increase the probability that infected carcasses are deposited in habitats where moisture and shade favour slower decomposition and prolonged environmental contamination.

Nevertheless, wetlands should be interpreted as a landscape-level indicator rather than as a direct causal factor. Municipality-level data cannot capture fine-scale habitat use, exact carcass locations, or local transmission processes. Moist and shaded environments may prolong virus survival in carcasses and contaminated substrates, thereby supporting indirect transmission [9,10]. In this context, wetland coverage may reflect environmental conditions that increase the likelihood of local ASF persistence, especially when combined with carcass-mediated transmission.

The three possible mechanisms discussed above, carcass persistence in moist habitats, wild boar behavioural preference for wetland-associated areas, and indirect transmission via contaminated water or soil, cannot be distinguished analytically from municipality-level observational data and may act simultaneously. The associations between wetland coverage and ASF presence in the longitudinal GEE model remained significant after adjustment for surveillance year and hunting-related sampling effort. However, hunting bag size does not account for carcass search effort; therefore, differences in detection probability cannot be excluded as a contributing explanation.

### 4.3. Carcass Detection and Surveillance

While surveillance was not the main focus of this study, the interpretation of ASF detection patterns requires consideration of the surveillance context. Although most samples originated from hunted wild boar, the proportion of ASF-positive animals was much higher among found dead wild boar, especially in endemic municipalities. This pattern is consistent with previous evidence that passive surveillance is more efficient than active surveillance for detecting ASF in wild boar populations [16,23].

Carcass detection is also epidemiologically relevant because infected carcasses can contaminate the environment and contribute to indirect virus transmission. Previous studies have shown that carcass-mediated transmission may support ASF persistence in wild boar populations, especially when environmental conditions allow prolonged virus survival [10,24,25]. Therefore, carcass detection in this study should be interpreted mainly as part of the surveillance and detection process, while environmental conditions may help explain differences in ASF persistence between municipalities.

### 4.4. Interpretation of Hunting Bag Data

No significant differences in the number of hunted wild boar were observed between the three municipality groups. This suggests that ASF status was not reflected in a measurable difference in hunting output at the municipality level. However, hunting bag data should be interpreted cautiously, as they do not directly represent wild boar density and may be influenced by hunting effort, hunter participation, accessibility, management regulations, and reporting practices. Previous assessments have emphasised that hunting data are among the most widely available indicators for wild boar monitoring, but they are often not accompanied by standardised measures of hunting effort [26,27].

Similar hunting bag numbers between municipality groups may therefore reflect surveillance and management-related factors rather than comparable wild boar abundance. The fact that hunting bags were not lower in endemic municipalities suggests that any reduction in wild boar numbers due to ASF-related mortality or control measures was not clearly reflected in municipality-level hunting data. In Lithuania, previous analyses similarly showed that the effects of intensified hunting could not be clearly separated from ASF-induced population decline [16]. Given the high mortality associated with ASF and the intensified hunting in ASF-affected areas, a reduction in local wild boar abundance could be expected, especially after longer periods of virus circulation. However, ASF-affected zones do not necessarily correspond to municipality boundaries and may extend across several municipalities. Therefore, the absence of lower hunting bags in endemic municipalities may reflect a combination of intensified hunting effort, ASF-related mortality, spatial mismatch between management zones and administrative units, and possible compensatory population processes. These mechanisms could not be distinguished using the available municipality-level data and should be investigated in future studies [28]. In the context of ASF persistence, these findings support the interpretation that regular hunting alone may be insufficient to interrupt virus circulation, especially when carcass-mediated transmission and environmental persistence contribute to disease maintenance [29,30].

### 4.5. Limitations

Several limitations should be considered when interpreting the findings of this study. First, the analysis was conducted at the municipality level, representing a relatively broad spatial scale compared to wild boar movement ecology. The municipalities included in the analysis ranged in size from 79 to 2216 km^2^, whereas reported wild boar home ranges are considerably smaller and may vary depending on habitat structure, season, sex, and population density [31,32,33]. For example, home ranges of wild boar have been reported to range from 4–11 km^2^ to nearly 90 km^2^ [33]. This spatial scale may mask local heterogeneity in ASF dynamics, including carcass distribution, wild boar movement, and localised transmission. In addition, ASF spread is spatially structured, and spatial autocorrelation between neighbouring municipalities was not explicitly modelled, which should be considered when interpreting municipality-level associations. Neighbouring municipalities may share environmental conditions and epidemiologically linked ASF detections. Consequently, the observed associations may partly reflect regional patterns of disease spread and detection rather than independent effects of local environmental characteristics. Moreover, carcass locations represent only the place of detection and do not reflect the full home range or movement patterns of individual wild boar. In addition, the small number of municipalities with no ASF detected (*n* = 5) and the unequal size of municipality groups should be considered when interpreting group comparisons, especially those involving areas with no ASF detected. The negative binomial models did not explicitly account for correlation between annual observations within the same municipality. This may affect standard errors and confidence intervals; therefore, statistical significance in these models should be interpreted cautiously.

Second, the use of surveillance-based data may have introduced detection bias, as reported ASF cases depend not only on infection occurrence but also on sampling effort, carcass search efficiency, and reporting practices. Hunting bag size reflects hunting-related sampling effort but does not account for the intensity or effectiveness of carcass searches, which are especially relevant because most ASF-positive animals were detected through passive surveillance. Although carcass search requirements were applied nationally, actual search effort and detection probability may have varied with accessibility, vegetation cover, and local surveillance practices. Municipality-level carcass search effort could not be quantified; therefore, the observed environmental associations may partly reflect differences in detection probability rather than infection occurrence alone.

The operational definition of endemic status, based on detections in at least three consecutive years, cannot distinguish uninterrupted local transmission from repeated introductions. Both processes may contribute to prolonged ASF occurrence and require sustained control measures. Associations with endemic status should therefore be interpreted as associations with prolonged ASF detection, rather than as evidence of factors maintaining uninterrupted local transmission.

Third, environmental variables were assessed as aggregated proportions. Forest cover was treated as time-varying, whereas wetland coverage was considered static over the study period. The analysis did not include a full land-cover composition assessment or spatial configuration metrics, such as habitat and landscape fragmentation.

Finally, some potentially relevant variables, including direct estimates of wild boar density, soil characteristics, and microclimate, were not included due to data limitations. Future studies using higher-resolution spatial data and dynamic environmental variables could provide a more detailed understanding of ASF persistence mechanisms.

### 4.6. Implications

These findings have implications for ASF surveillance and control in endemic wild boar populations, particularly in Lithuania, where ASF has affected wild boar for more than a decade. The associations with forest cover and wetland coverage suggest that environmental conditions should be considered when interpreting ASF patterns and planning surveillance. In particular, moist and wetland-associated habitats may require more attention, as these areas may support carcass persistence and indirect virus transmission.

The relationship between forest cover and detected ASF case counts also indicates that landscape structure and habitat accessibility may influence surveillance outcomes. Strategies focused only on host population reduction may therefore be insufficient if environmental and spatial factors affecting virus persistence and carcass detectability are not considered.

The findings also support cautious interpretation of hunting data. Similar hunting bags between municipality groups do not necessarily indicate comparable wild boar densities or disease dynamics. Control approaches should therefore combine population management with systematic carcass detection and removal, especially in areas where environmental conditions may favour virus persistence.

## 5. Conclusions

Environmental factors were associated with ASF detection and persistence patterns in wild boar populations across Lithuanian municipalities during 2016–2022. Wetland coverage showed consistent positive associations with endemic status, detected case counts, and ASF presence over time, whereas forest cover was negatively associated with detected case counts but not significantly associated with endemic status or ASF presence. These findings highlight the importance of considering landscape characteristics alongside surveillance processes when interpreting municipality-level ASF patterns. The high proportion of ASF-positive animals among wild boar found dead further supports the value of carcass detection in surveillance. Overall, the findings support targeted surveillance and control approaches that consider environmental conditions and combine population management with systematic carcass detection and removal.

## Figures and Tables

**Table 1:** Variables included in the statistical analyses.

Variable Group	Variable	Definition/Unit	Use in Analysis
Outcome/grouping	ASF status group	Municipality-level category: endemic, non-endemic, or No ASF detected	Group comparisons
	ASF-positive wild boar cases	Number of PCR-positive wild boar per municipality-year and total per municipality	Group comparisons; box plot; NB regression
	ASF presence/absence	Binary variable indicating whether ASF was detected in a municipality-year	GEE model
Surveillance	Hunted wild boar	Number of hunted wild boar tested per municipality-year	Group comparison; correlation; adjustment
	Found dead wild boar	Number of found dead wild boar tested for ASFV	Group comparison
	ASF-positive found dead wild boar	Number of PCR-positive found dead wild boar	Group comparison
Environmental	Forest cover	Percentage of municipality area covered by forest; annual variable for 2016–2022	Negative binomial GLM; GEE model; logistic regression
	Wetland coverage	Percentage of municipality area covered by wetlands; time-invariant variable	Group comparison; negative binomial GLM; GEE; logistic regression
Spatial	Municipality area	Municipality area in km^2^	Offset in NB regression
	River density	Total river length per municipality area, km/km^2^	Exploratory analysis
Border-related	Country border presence	Binary variable indicating whether a municipality bordered another country	Chi-square test
	Border country	Categorical variable indicating the name of the bordering country	Chi-square test
Demographic	Human population size and density	Total, urban, and rural population indicators at municipality level	Exploratory correlation
Domestic pig ASF	Domestic pig ASF cases	Number of ASF outbreaks/cases in domestic pigs per municipality-year	Exploratory correlation
Temporal	Detection year	Year of ASF detection/surveillance year, 2016–2022	Model adjustment; temporal analysis

Note: ASF = African swine fever; ASFV = African swine fever virus; GLM = generalised linear model; GEE = generalised estimating equations; NB = negative binomial. Municipality-year refers to one municipality in one study year.

**Table 2:** Annual hunted wild boar numbers and their correlation with ASF-positive hunted wild boar cases, 2016–2022.

Year	Hunted Wild Boar	ASF-Positive Hunted Wild Boar	Spearman’s ρ	*p*-Value
2016	33,902	52	0.026	0.862
2017	30,991	283	0.239	0.099
**2018**	**20,552**	**247**	**0.557**	**<0.001**
**2019**	**10,485**	**122**	**0.374**	**0.007**
**2020**	**6930**	**20**	**0.380**	**0.006**
**2021**	**14,386**	**101**	**0.440**	**0.001**
**2022**	**20,234**	**47**	**0.292**	**0.040**

Note: ρ, Spearman’s rank correlation coefficient. Correlations were calculated separately for each year. Statistically significant correlations are shown in bold.

**Table 3:** Summary of regression models assessing associations between environmental variables and ASF occurrence in Lithuanian wild boar, 2016–2022.

Model	Outcome	Environmental Predictor	B	OR/IRR (95% CI)	*p*-Value	Adjustment Variables
Univariable logistic regression	Endemic status	Forest cover (%)	0.014	OR 1.01 (0.996–1.032)	0.127	None
Univariable logistic regression	Endemic status	Wetland coverage (%)	0.452	OR 1.57 (1.274–1.939)	<0.001	None
Negative binomial regression—forest model	Annual ASF-positive case count	Forest cover (%)	–0.040	IRR 0.961 (0.936–0.986)	0.002	Year; ln(hunted wild boar)
Negative binomial regression—wetland model	Annual ASF-positive case count	Wetland coverage (%)	0.251	IRR 1.285 (1.055–1.565)	0.013	Year
Negative binomial regression—joint model	Annual ASF-positive case count	Forest cover (%)	–0.043	IRR 0.958 (0.946–0.970)	<0.001	Year; ln(hunted wild boar); wetland cover
Negative binomial regression—joint model	Annual ASF-positive case count	Wetland coverage (%)	0.128	IRR 1.137 (1.023–1.261)	0.017	Year; ln(hunted wild boar); forest cover
Logistic GEE—forest model	Annual ASF absence	Forest cover (%)	–0.025	OR 0.975 (0.944–1.009)	0.155	Year; ln(hunted wild boar)
Logistic GEE—wetland model	Annual ASF presence	Wetland coverage (%)	0.278	OR 1.32 (1.002–1.739)	0.048	Year ln(hunted wild boar)

Note: B = regression coefficient; OR = odds ratio; IRR = incidence rate ratio; CI = confidence interval; GEE = generalised estimating equations; ln = natural logarithm. All negative binomial and GEE models included 350 municipality-year observations from 50 municipalities during 2016–2022. Environmental effect estimates are expressed per one percentage-point increase in forest cover or wetland coverage. B is expressed on the log-odds scale for logistic models and the log scale for negative binomial models. All negative binomial models included ln(municipality area) as an offset. IRRs and their confidence intervals were calculated by exponentiating the reported coefficients and confidence limits and are approximate due to rounding. GEE models accounted for repeated observations within municipalities, using an independent working correlation structure for the forest model and an exchangeable structure for the wetland model. The forest GEE model estimated the odds of ASF absence, whereas the wetland GEE model estimated the odds of ASF presence. Adjustment variables are listed separately for each model.

## Data Availability

The epidemiological data analysed in this study were provided by the State Food and Veterinary Service of Lithuania with permission for use in this study. These data are not publicly available, as the permission granted does not cover public sharing or redistribution. Access requires separate authorization from the State Food and Veterinary Service of Lithuania. Other data sources used in this study are identified in Section 2.

## Abbreviations

The following abbreviations are used in this manuscript:

ASF	African swine fever
ASFV	African swine fever virus
CI	Confidence interval
CSF	Classical swine fever
df	Degrees of freedom
DNA	Deoxyribonucleic acid
GEE	Generalised estimating equations
GLM	Generalised linear model
IRR	Incidence rate ratio
NB	Negative binomial
OR	Odds ratio
PCR	Polymerase chain reaction
SD	Standard deviation
SE	Standard error

## Appendix A

**Table A1:** Annual numbers of hunted and found dead wild boar tested for ASFV and ASF-positive cases in the 50 Lithuanian municipalities included in the analysis, 2016–2022.

Year	Hunted Wild Boar	Found Dead Wild Boar	Total Tested	ASF-Positive Cases	ASF-Positive Hunted, *n* (%)	95% CI (%)	ASF-Positive Found Dead, *n* (%)	95% CI (%)
2016	33,902	750	34,652	421	52 (0.15%)	0.12–0.20	369 (49.20%)	45.64–52.77
2017	30,991	2612	33,603	2348	283 (0.91%)	0.81–1.03	2065 (79.06%)	77.46–80.58
2018	20,552	2197	22,749	1984	247 (1.20%)	1.06–1.36	1737 (79.06%)	77.31–80.71
2019	10,485	408	10,893	332	122 (1.16%)	0.98–1.39	210 (51.47%)	46.63–56.28
2020	6930	118	7048	21	20 (0.29%)	0.19–0.45	1 (0.85%)	0.15–4.64
2021	14,386	131	14,517	162	101 (0.70%)	0.58–0.85	61 (46.56%)	38.24–55.08
2022	20,234	112	20,346	97	47 (0.23%)	0.17–0.31	50 (44.64%)	35.77–53.87
Total	137,480	6328	143,808	5365	872 (0.63%)	0.59–0.68	4493 (71.00%)	69.87–72.11

**Table A2:** Municipality area, ASF status group, total ASF-positive wild boar cases, and ASF-positive case density in the municipalities included in the analysis, 2016–2022.

Municipality	ASF Status Group	Area, km^2^	Total ASF-Positive Cases, 2016–2022	ASF-Positive Cases per 100 km^2^
Akmene	Non-endemic	844	84	9.95
Alytus	Endemic	1403	60	4.28
Anyksciai	Endemic	1764	764	43.31
Birstonas	Non-endemic	122	4	3.28
Birzai	Endemic	1476	128	8.67
Druskininkai	Endemic	453	9	1.99
Elektrenai	Endemic	509	126	24.75
Ignalina	Endemic	1441	42	2.91
Jonava	Endemic	943	132	14.00
Joniskis	Non-endemic	1151	26	2.26
Jurbarkas	Non-endemic	1506	237	15.74
Kaisiadorys	Endemic	1087	87	8.00
Kalvarija	No ASF detected	440	0	0.00
Kaunas	Endemic	1495	207	13.85
Kazlu Ruda	Non-endemic	555	11	1.98
Kedainiai	Endemic	1677	208	12.40
Kelme	Non-endemic	1705	11	0.65
Klaipeda	No ASF detected	1340	0	0.00
Kupiskis	Endemic	1080	25	2.31
Lazdijai	Endemic	1306	182	13.94
Marijampole	Non-endemic	755	36	4.77
Mazeikiai	Endemic	1219	67	5.50
Moletai	Endemic	1367	131	9.58
Neringa	No ASF detected	90	0	0.00
Pakruojis	Non-endemic	1315	91	6.92
Palanga	No ASF detected	79	0	0.00
Panevezys	Endemic	2177	595	27.33
Pasvalys	Endemic	1289	128	9.93
Plunge	Endemic	1105	15	1.36
Prienai	Endemic	1032	36	3.49
Radviliskis	Endemic	1634	69	4.22
Raseiniai	Endemic	1573	45	2.86
Rietavas	No ASF detected	586	0	0.00
Rokiskis	Endemic	1806	70	3.88
Sakiai	Non-endemic	1454	69	4.75
Salcininkai	Endemic	1493	30	2.01
Siauliai	Non-endemic	1807	97	5.37
Silute	Non-endemic	1714	6	0.35
Sirvintos	Endemic	906	434	47.90
Skuodas	Endemic	911	75	8.23
Svencionys	Endemic	1691	15	0.89
Taurage	Non-endemic	1179	25	2.12
Telsiai	Endemic	1439	57	3.96
Trakai	Endemic	1207	41	3.40
Ukmerge	Endemic	1395	545	39.07
Utena	Endemic	1230	73	5.93
Varena	Endemic	2216	97	4.38
Vilkaviskis	Non-endemic	1262	1	0.08
Vilnius	Endemic	2129	61	2.87
Zarasai	Endemic	1331	113	8.49

Note: ASF = African swine fever. ASF-positive case density was calculated as the total number of PCR-positive wild boar cases during 2016–2022 divided by municipality area and multiplied by 100.

**Table A3:** Additional exploratory analyses of selected municipality-level variables.

Variable	Year	Test	Spearman’s ρ/Test Statistic	*p*-Value
River density		Kruskal–Wallis H test across ASF status groups	H = 2.871; df = 2	0.238
Domestic pig ASF cases	2016	Spearman	ρ = 0.413	0.003
Domestic pig ASF cases	2017	Spearman	ρ = 0.588	<0.001
Domestic pig ASF cases	2018	Spearman	ρ = 0.464	0.001
Domestic pig ASF cases	2019	Spearman	ρ = 0.499	<0.001
Domestic pig ASF cases	2020	Spearman	ρ = 0.459	0.001
Domestic pig ASF cases	2021	Spearman	Not calculated	N/A
Domestic pig ASF cases	2022	Spearman	ρ = 0.392	0.005
Total human population	2016	Spearman	ρ = 0.180	0.211
Total human population	2017	Spearman	ρ = 0.086	0.555
Total human population	2018	Spearman	ρ = 0.213	0.138
Total human population	2019	Spearman	ρ = 0.272	0.056
Total human population	2020	Spearman	ρ = 0.109	0.451
Total human population	2021	Spearman	ρ = 0.127	0.381
Total human population	2022	Spearman	ρ = 0.060	0.681
Rural human population	2016	Spearman	ρ = 0.195	0.175
Rural human population	2017	Spearman	ρ = 0.066	0.648
Rural human population	2018	Spearman	ρ = 0.223	0.120
Rural human population	2019	Spearman	ρ = 0.262	0.066
Rural human population	2020	Spearman	ρ = 0.133	0.356
Rural human population	2021	Spearman	ρ = 0.158	0.273
Rural human population	2022	Spearman	ρ = 0.162	0.262

Note: Spearman correlations were calculated using same-year municipality-level values. The 2021 correlation for domestic pig ASF cases was not calculated because no domestic pig ASF outbreaks were reported in that year. N/A = not applicable.
